# Bilateral ovarian fibromas as the sole manifestation of Gorlin syndrome in a 22-year-old woman: a case report and literature review

**DOI:** 10.1186/s13000-023-01406-9

**Published:** 2023-10-31

**Authors:** Menghan Zhu, Jun Li, Jie Duan, Jing Yang, Weiyong Gu, Wei Jiang

**Affiliations:** 1 Department of Gynecology, Obstetrics and Gynecology Hospital, Fudan University, Shenyang Road 128, Shanghai, 200090 China; 2 Department of Pathology, Obstetrics and Gynecology Hospital, Fudan University, Shanghai, 200090 China

**Keywords:** Gorlin syndrome, NBCCS, Ovarian fibroma, Genetic counseling, Ovarian preservation

## Abstract

**Background:**

Nevoid basal cell carcinoma syndrome (NBCCS, Gorlin syndrome) is a rare autosomal dominantly inherited disorder that is characterized by multisystem disorder such as basal cell carcinomas, keratocystic odontogenic tumors and skeletal abnormalities. Bilateral and/or unilateral ovarian fibromas have been reported in individuals diagnosed with NBCCS.

**Case presentation:**

A 22-year-old female, presented with low back pain, and was found to have bilateral giant adnexal masses on pelvic ultrasonography, which had been suspected to be malignant ovarian tumors. Positron emission tomography/computed tomography showed multiple intracranial calcification and skeletal abnormalities. The left adnexa and right ovarian tumor were resected with laparotomy, and pathology revealed bilateral ovarian fibromas with marked calcification. We recommended the patient to receive genetic testing and dermatological examination. No skin lesion was detected. Germline testing identified pathogenic heterozygous mutation in *PTCH1* (*Patched1*).

**Conclusions:**

The possibility of NBCCS needs to be considered in patients with ovarian fibromas diagnosed in an early age. Skin lesions are not necessary for the diagnosis of NBCCS. Ovarian fibromas are managed with surgical excision with an attempt at preserving ovarian function. Follow-up regime and counseling on options for future fertility should be offered to patients.

## Background

Ovarian fibroma is the most common sex cord-stromal tumor, accounting for 4% of all ovarian tumors [1]. It can occur at any age, but it is most common after puberty (mean 30.6 years, range 16– 45 years) and seldom occurs before 30 years old. It is often misdiagnosed preoperatively as uterine fibroid due to its solid nature and similar clinical and ultrasound findings. Ovarian fibromas have been reported in 15–25% of patients diagnosed with NBCCS, 75% of those being bilateral [2–6]. NBCCS, or basal cell nevus syndrome (Gorlin syndrome) is a rare autosomal dominantly inherited disorder with a prevalence of 1 in 57,000 to 1 in 164,000 [2]. It is characterized by multisystem disorder such as basal cell carcinomas (BCCs), keratocystic odontogenic tumors (KCOTs), palmar and plantar pits, and a range of skeletal and developmental abnormalities [7, 8]. Early diagnosis and treatment of NBCCS, as well as family screening and genetic counseling, are essential as it may be associated in 10% of the patients with aggressive BCCs and malignant neoplasia. Most of the reported cases were diagnosed with NBCCS before the discovery of ovarian fibromas. It is rare to diagnose NBCCS during the accidental discovery of ovarian fibromas in adult women. Herein, we report a 22-year-old female with bilateral calcified ovarian fibromas associated with accidentally revealed multiple intracranial calcification and scoliosis, and has no remarkable individual or family history. Germline testing identified pathogenic heterozygous mutation in *PTCH1.*

## Case presentation

**Fig. 1:**
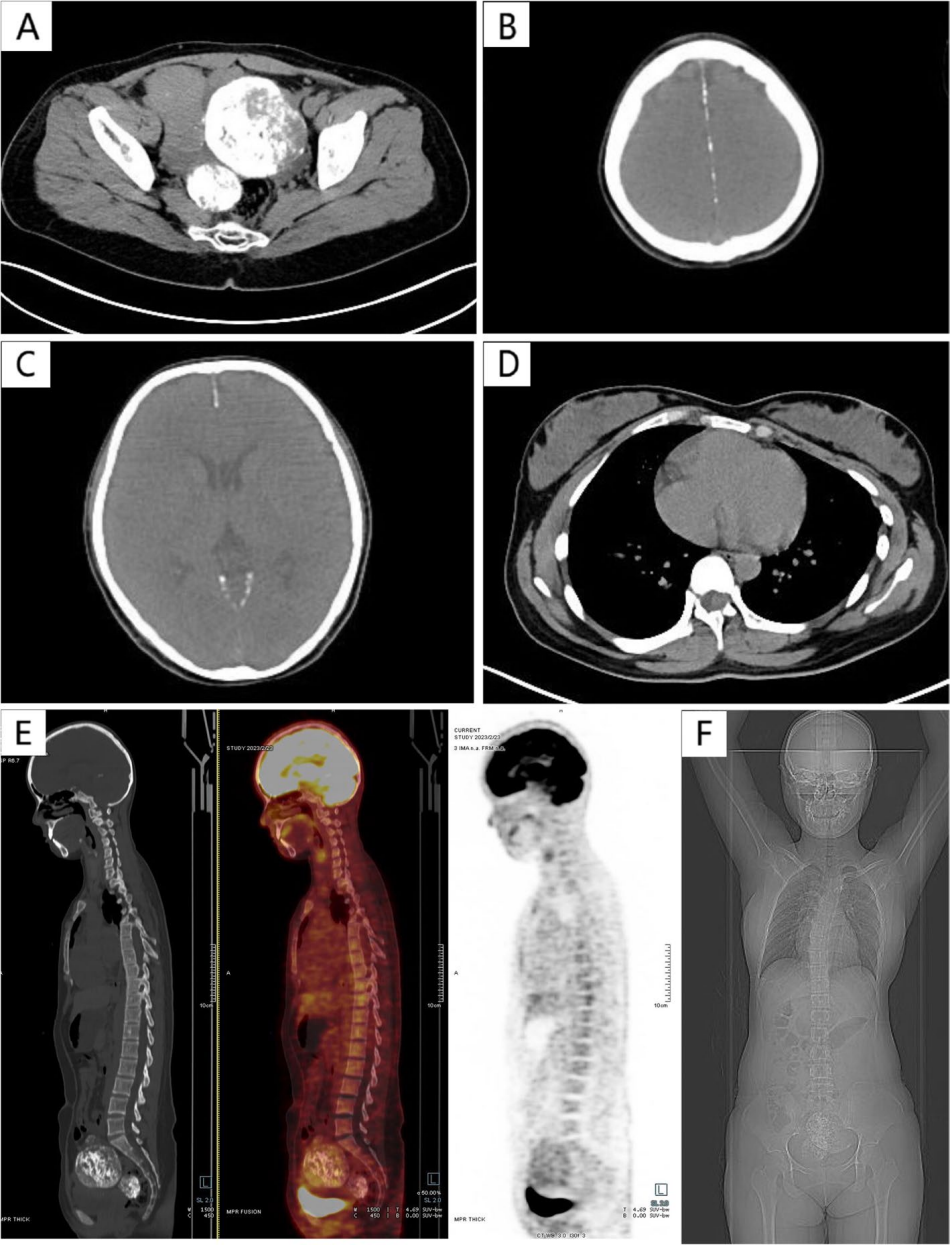
PET/CT imaging. **A** Axial CT showing multiple high-density nodular masses in the lower abdomen and pelvic cavity with multiple calcifications. **B** Axial CT showing extensive nodular calcifications in the falx cerebri. **C** Axial CT showing extensive nodular calcifications in the tentorium cerebelli. **D** Axial CT showing asymmetry in the thorax with a slight collapse in the left side. **E** Mildly increased uptake of FDG within the pelvic masses. **F** X-ray posteroanterior, chest view, showing scoliosis of thoracic spine

The patient was a 22-year-old Mongolian female without sexual experience. She had a height of 174 cm and weight of 65 kg. Her menstrual cycle has been regular since her menarche at the age of 12. There was no remarkable individual or family history. She went to a hospital due to persistent low back pain for 1 week. Abdominal CT examination revealed multiple low-density masses with calcification in bilateral adnexal areas, which were presumed as uterine fibroids originate from the broad ligament. The patient was then transferred to our hospital for treatment. On general examination, she was of average build and nutrition. There was no pallor, and her vitals were stable. She had no sign of androgen excess. Abdominal examination revealed a firm, solid mass corresponding to 16 weeks of gestation. The edge of the mass was not palpable. Transabdominal ultrasound revealed a normal-sized uterus and bilateral adnexal masses, which were presumed as ovarian tumors with malignant potential. In order to evaluate the primary and metastatic lesions through a whole-body tomography and metabolic imaging, she underwent a positron emission tomography/computed tomography (PET/CT) examination. PET/CT showed the following characteristics (Fig. 1): 1) multiple high-density nodular masses in the lower abdomen and pelvic cavity with multiple calcifications,2) extensive nodular calcifications in the falx cerebri and tentorium cerebelli. The 18-fluorodeoxyglucose (FDG) uptake of the masses was mildly increased, with maximum standardized uptake value of 3.9. Meanwhile, it was identified asymmetry in her bilateral thorax with a slight collapse in her left side, and scoliosis of thoracic spine. Her serum calcium and phosphorus were within the normal range. Serum cancer antigen 125 (CA125) slightly increased to 35.10U/ml (Normal: ≤ 35U/ml), while human epididymis protein 4 (HE4) was 29.2 pmol/l and serum inhibin A was 7.4 pg/ml both within the normal range. We decided the patient be indicated for the surgery.

**Fig. 2:**
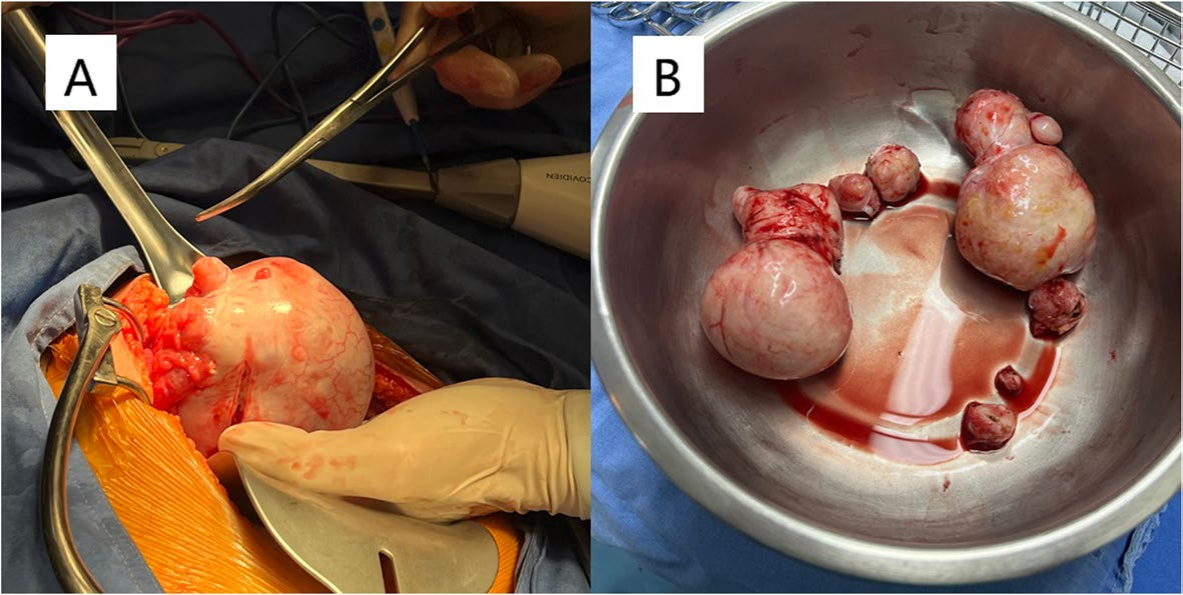
Gross images at laparotomy. **A** The left ovary presented as a white solid mass of 12 cm in diameter, with bumps of varying sizes and rich blood vessels visible on the surface. **B** The nodular fibroma of the right ovary

**Fig. 3:**
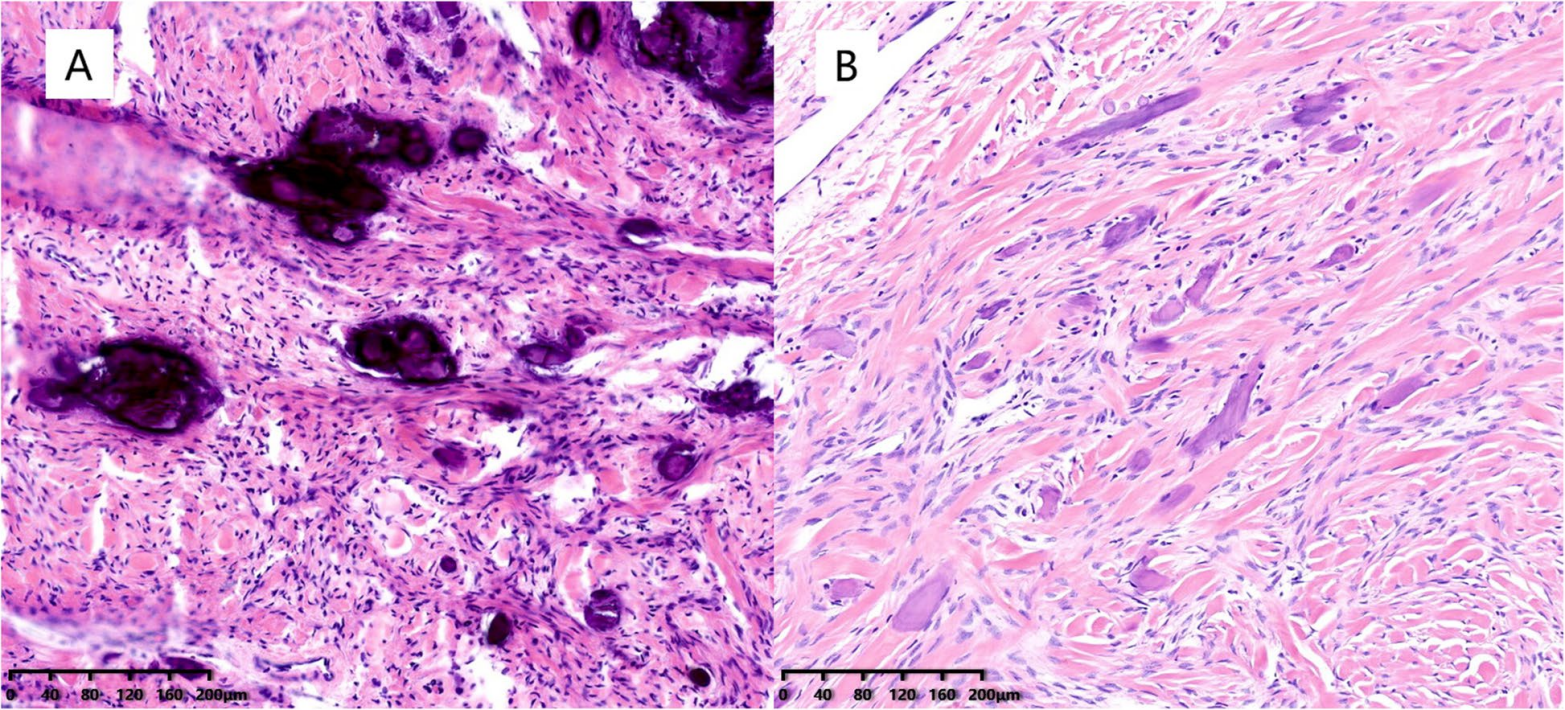
Representative image. The undecalcified (**A**) and decalcified (**B**) ovarian fibromas, showing benign spindle cells and calcification with no atypia (hematoxylin and eosin; magnification 100 ×)

We made a 15-cm incision in the lower abdomen. Intraoperative findings on laparotomy showed that there was no uterine myoma or ascites. Extremely rigid tumors were found in both ovaries. The left ovary presented as a white solid mass of 12 cm in diameter in irregular shape, with bumps of varying sizes and rich blood vessels visible on the surface. No obvious normal ovarian tissue was found in the left ovary. The right ovarian tumor presented as a white solid mass of 8 cm in diameter with irregular shape and multiple nodules (Fig. 2). Normal ovarian tissue of the right ovary was observed. No abnormality was found during exploration of the pelvis and abdomen. She underwent left salpingo-oophorectomy and right ovarian-sparing tumor resection. Microscopically, the bilateral tumors demonstrated spindled cells and frequent calcifications within a collagenous background, consistent with an ovarian fibroma (Fig. 3).

**Fig. 4:**
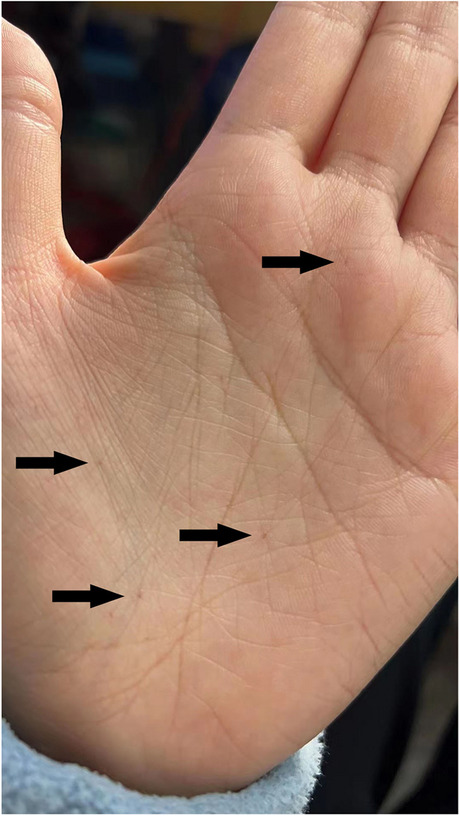
Dyskeratotic palmar pits (arrows)

The patient reported regular monthly menses after surgery. Although she did not report any skin lesion, we reviewed the imaging and pathology findings and considered the possibility of NBCCS. We recommended her to enhance sun protection in daily life, and further asked for some additional information. She reported that she had no relatives of diagnosed NBCCS. Her mother reported no abnormality in her birth, growth, vision/hearing, dentition, and development. She reported that she had multiple palmer pits which appear to be more obvious after bath (Fig. 4).

**Fig. 5:**
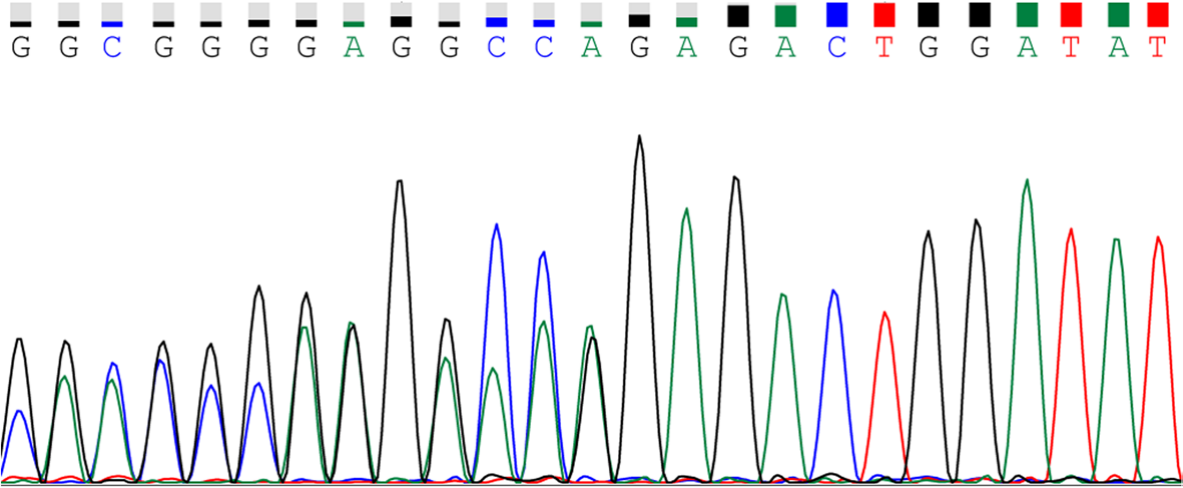
Sanger sequencing peak plot

We strongly recommended the patient to undergo a full dermatologic examination and to receive genetic testing and counseling. No basal cell carcinoma was found in dermatologic examination. The patient underwent germline testing for *PTCH1, PTCH2, SUFU, SMO, GL1*, and *GL2* gene. A heterozygous variation of c.1818delG was detected in *PTCH1* (Fig. 5). This mutation is a frame shift mutation, which is a loss of function mutation. Then we provided follow-up regime to the patient based on a guideline of the British Association of Dermatologists for the clinical management of NBCCS [9], and we also recommended her parents to receive genetic testing in order to providing presymptomatic screening.

Informed written consent was obtained from the patient for publication of this case report and accompanying images.

## Discussion and conclusions

Gorlin syndrome, also known as NBCCS or basal cell nevus syndrome (BCNS), is a heritable cancer predisposition syndrome with an autosomal dominant pattern of inheritance. It is characterized most strikingly by development of cutaneous BCCs from an early age. Gorlin and Goltz firstly described this syndrome that included multiple BCCs, jaw cysts, and bifid ribs in 1960 [10]. The estimated prevalence is 1 in 57,000 to 1 in 164,000 and no sex predilection has been observed. Affected individuals can have multiple phenotypic abnormalities, with characteristic facial features described in over 50% of individuals that may include coarse facial appearance, macrocephaly, and hypertelorism. Multiple jaw KCOTs are seen in 75%–90% of the patients with NBCCS. Other well-recognized clinical features include dyskeratotic palmar and plantar pits, rib and spine abnormalities, and early calcification of the falx cerebri [4, 7, 8, 11].

Individuals with NBCCS are at risk for developing both benign and malignant neoplasms. Multiple nevoid BCCs over the nose, eyelids, cheeks and elsewhere are often an early sign. Skin lesions often appear in puberty, in some cases occurring earlier in childhood. However, there is no clear genotype–phenotype correlation for the timing or number of BCCs that develop [2]. Approximately 5% of individuals with NBCCS develop medulloblastoma at a mean age of 2 years old. Cardiac fibromas may develop in infants and ovarian fibromas in adolescent women. Ovarian fibromas have been reported in 15–25% of patients diagnosed with NBCCS, 75% of those being bilateral. While fibromas with NBCCS are typically diagnosed between 16 and 45 years of age, the reported diagnosed age is as early as 3.5 years old [12].

In prior case reports, the majority were diagnosed with NBCCS before the discovery of ovarian fibromas (Table 1). Due to characteristic features and visible skin lesions appearing from an early age, it is rare to diagnose NBCCS during the accidental discovery of ovarian fibromas in adult women.

**Table 1:** Ovarian fibromas with NBCCS published in literature

Author	Year	Age (year)	Race/ethni-city/nationality	Symptom	History	Ovarian fibroma	Previously diagnosed with NBCCS
Zhu	Current case	22	Mongolian	Low back pain	None	Bilateral	No (Diagnosed at age 22 years by ovarian fibromas, intracranial calcification, skeletal abnormalities and palmar pits. Germline testing identified pathogenic heterozygous mutation in *PTCH1*.)
Morse [13]	2011	15	Caucasian	Irregular menses	Medulloblastoma at 6 months, multiple KCOTs at age 7 years	Bilateral	Yes
Seracchioli [3]	2001	22	Italian	Irregular menses	KCOTs resection 5 times between age 9–16 years, BCCs at age 16 years	Bilateral	Yes
Aram [14]	2009	22	Iranian	Irregular menses	KCOTs at age 12 years; facial dysmorphism	Bilateral	Yes
Finch [15]	2012	22	Caucasian	Irregular menses	KCOTs removed at ages 7, 13 and 20	Unilateral	Yes
Pirschner [6]	2012	20	Brazilian	Abdominal swelling	KCOTs at age 10 years; facial asymmetry, micrognathism	Bilateral	Yes
Osaku [5]	2021	24	Unknown	None	KCOTs resection at the age of 18 and 20 years, palmar /plantar pits	Unilateral	Yes
Jimbo [16]	2014	6	Japanese	Abdominal distension	Macrocephaly at 4 months, medulloblastoma at age 4 years	Unilateral	Yes
Johnson [12]	1986	3.5	Black	Unknown	None	Bilateral	No (Diagnosed at age 4 years when basal cell tumors and palmar/plantar pits were noted on examination.)
Higashi-moto [17]	2022	5	Japanese	Ovarian torsion	None	Bilateral	No (Diagnosed at age 5 years by detecting de novo germline variants in *PTCH1.)*

The diagnostic criteria are described in Table 2, with either two major criteria or one major and two minor criteria being required for confirmation of diagnosis [11]. Our case meets two major (palmar pits, calcification of the falx cerebri) and two minor (ovarian fibroma, skeletal malformations) diagnostic criteria for NBCCS.

**Table 2:** Diagnostic criteria for NBCCS [11]

Major criteria
1. BCCs before 20 years of age or excessive numbers of BCCs out of proportion to prior sun exposure and skin type
2. KCOTs before 20 years of age
3. Palmar or plantar pits
4. Lamellar calcification of the falx cerebri
5. Medulloblastoma, typically desmoplastic
6. First degree relative with NBCCS
Minor criteria
1. Rib abnormalities
2. Other specific skeletal malformations and radiologic changes (i.e., vertebral anomalies, kyphoscoliosis, short fourth metacarpals, postaxial polydactyly)
3. Macrocephaly
4. Cleft lip or palate
5. Ovarian or cardiac fibroma
6. Lymphomesenteric cysts
7. Ocular abnormalities (i.e., strabismus, hypertelorism, congenital cataracts, glaucoma, coloboma)

Management of ovarian fibroma involves conservative excision with an attempt at ovarian functional preservation. Preservation of the normal ovarian tissue is always recommended [3]. In our case, due to the absence of normal ovarian tissue in her left ovary, we performed left salpingo-oophorectomy and right ovarian-sparing tumor resection for ovarian functional preservation. Although there have been few cases reported, it is observed that ovarian fibromas rarely recur after surgery [3]. Although no data exist about the fertility potential of females with NBCCS, surgical resection may lead to compromised fertility. Females with NBCCS should be counseled about techniques available to maintain future reproductive options.

NBCCS is associated with germline mutations in components of the Sonic Hedgehog pathway, including Patched1 (*PTCH1*) and Suppressor of fused (*SUFU*) [18, 19]. Most mutations occur as loss of function mutations in the *PTCH1* gene located on chromosome 9q22.3. Heterozygous germline mutations in *PTCH1* have been detected in the majority of individuals with NBCCS. Less frequently, germline mutations in *SUFU* are observed. About 75% of individuals with NBCCS have an affected parent, with the remainder presumably due to de novo germline variants [4]. Developmental deficits and malignancies associated with NBCCS are thought to develop via a two-hit mechanism [8]. For individuals with an identified mutation, preimplantation genetic diagnosis (PGD) may be an option to avoid passing the genetic mutation to biologic offspring, although there are currently no reports of PGD for NBCCS. In our case, germline testing identified pathogenic heterozygous mutation in *PTCH1.*

This case demonstrates a rare case of NBCCS diagnosed during the accidental discovery of ovarian fibromas in an adult woman. The possibility of NBCCS needs to be considered in patients with ovarian fibromas diagnosed in an early age. It is effective to manage ovarian fibromas with ovarian-sparing surgical excision. It is recommended that patients with suspected NBCCS undergo dermatological examinations, genetic testing and counseling to early diagnose and treat BCCs. Follow-up regime and counseling on options for future fertility should be offered to patients.

## Data Availability

The raw data contains patient’s names, phone numbers and family addresses. According to our informed consent, we may not publish the raw data which may reveal the personal information of the patient. However, data are available from the corresponding author on reasonable request.

## Abbreviations

NBCCS: Nevoid basal cell carcinoma syndrome
BCNS: Basal cell nevus syndrome
BCCs: Basal cell carcinomas
KCOTs: Keratocystic odontogenic tumors
PET/CT: Positron emission tomography/computed tomography
FDG: 18-Fluorodeoxyglucose
CA125: Cancer antigen 125
HE4: Human epididymis protein 4
PTCH1: Patched1
SUFU: Suppressor of fused
PGD: Preimplantation genetic diagnosis

## References

[1] Chung BM, Park SB, Lee JB, Park HJ, Kim YS, Oh YJ (2015). Magnetic resonance imaging features of ovarian fibroma, fibrothecoma, and thecoma. Abdom Imaging.

[2] Kimonis VE, Goldstein AM, Pastakia B, Yang ML, Kase R, DiGiovanna JJ, Bale AE, Bale SJ (1997). Clinical manifestations in 105 persons with nevoid basal cell carcinoma syndrome. Am J Med Genet.

[3] Seracchioli R, Bagnoli A, Colombo FM, Missiroli S, Venturoli S (2001). Conservative treatment of recurrent ovarian fibromas in a young patient affected by Gorlin syndrome. Hum Reprod.

[4] Foulkes WD, Kamihara J, Evans D, Brugières L, Bourdeaut F, Molenaar JJ, Walsh MF, Brodeur GM, Diller L (2017). Cancer surveillance in Gorlin syndrome and rhabdoid tumor predisposition syndrome. Clin Cancer Res.

[5] Osaku D, Taniguchi F, Komatsu H, Wibisono H, Azuma Y, Harada T (2021). Calcified ovarian fibromas complicated with basal cell nevus syndrome. Gynecol Minim Invasive Ther.

[6] Pirschner F, Bastos PM, Contarato GL, Bimbato AC, Filho AC (2012). Gorlin syndrome and bilateral ovarian fibroma. Int J Surg Case Rep.

[7] Bale AE, Gailani MR, Leffell DJ (1994). Nevoid basal cell carcinoma syndrome. J Invest Dermatol.

[8] Bresler SC, Padwa BL, Granter SR (2016). Nevoid basal cell carcinoma syndrome (Gorlin syndrome). Head Neck Pathol.

[9] Verkouteren B, Cosgun B, Reinders M, Kessler PAWK, Vermeulen RJ, Klaassens M, Lambrechts S, van Rheenen JR, van Geel M, Vreeburg M, Mosterd K (2022). A guideline for the clinical management of basal cell naevus syndrome (Gorlin-Goltz syndrome). Br J Dermatol.

[10] Gorlin RJ, Goltz RW (1960). Multiple nevoid basal-cell epithelioma, jaw cysts and bifid rib. A syndrome N Engl J Med.

[11] Bree AF, Shah MR (2011). Consensus statement from the first international colloquium on basal cell nevus syndrome (BCNS). Am J Med Genet A.

[12] Johnson AD, Hebert AA, Esterly NB (1986). Nevoid basal cell carcinoma syndrome: bilateral ovarian fibromas in a 3 1/2-year-old girl. J Am Acad Dermatol.

[13] Morse CB, Mclaren JF, Roy D, Siegelman ES, Livolsi VA, Gracia CR (2011). Ovarian preservation in a young patient with Gorlin syndrome and multiple bilateral ovarian masses. Fertil Steril.

[14] Aram S, Moghaddam NA (2009). Bilateral ovarian fibroma associated with Gorlin syndrome. J Res Med Sci.

[15] Finch T, Pushpanathan C, Brown K, El-Gohary Y (2012). Gorlin syndrome presenting with a unilateral ovarian fibroma in a 22-year-old woman: a case report. J Med Case Rep.

[16] Jimbo T, Masumoto K, Urita Y, Takayasu H, Shinkai T, Uesugi T, Gotoh C, Sakamoto N, Sasaki T, Oto T, Fukushima T, Noguchi E, Nakano Y (2014). Nevoid basal cell carcinoma syndrome with a unilateral giant ovarian fibroma in a Japanese 6-year-old girl. Eur J Pediatr.

[17] Higashimoto T, Smith CH, Hopkins MR, Gross J, Xing D, Lee JW, Morris T, Bodurtha J (2022). Case report of bilateral ovarian fibromas associated with de novo germline variants in PTCH1 and SMARCA4. Mol Genet Genomic Med.

[18] Johnson RL, Rothman AL, Xie J, Goodrich LV, Bare JW, Bonifas JM, Quinn AG, Myers RM, Cox DR, Epstein EH, Scott MP (1996). Human homolog of patched, a candidate gene for the basal cell nevus syndrome. Science.

[19] Smith MJ, Beetz C, Williams SG, Bhaskar SS, O'Sullivan J, Anderson B, Daly SB, Urquhart JE, Bholah Z, Oudit D, Cheesman E, Kelsey A, McCabe MG, Newman WG, Evans DG (2014). Germline mutations in SUFU cause Gorlin syndrome-associated childhood medulloblastoma and redefine the risk associated with PTCH1 mutations. J Clin Oncol.

